# Open Dialogue as a cultural practice - critical perspectives on power obstacles when teaching and enabling this approach in current psychiatry

**DOI:** 10.3389/fpsyg.2022.1063747

**Published:** 2023-02-17

**Authors:** Sebastian von Peter, Katrin Eissing, Katharina Saliger

**Affiliations:** ^1^Medical School Brandenburg, Brandenburg an der Havel, Germany; ^2^Offener Dialogue e.V. Leipzig, Artist, Freelance, Leipzig, Germany; ^3^Mobile Krisenbegleitung Spitäler fmi AG Interlaken, Interlaken, Switzerland

**Keywords:** sect, monologue, difference, polyphonie, hierarchy, identity

## Abstract

Building on both therapeutic and organizational principles, adopting Open Dialogue (OD) calls various routines of the current mental health system into question, resulting in potential obstacles with implementation. This perspective paper aims to reflect on power relations as potential disruptive factors in enabling the OD approach in mental health care. Drawing on data from a small implementation study, followed by reflections from three perspectives, we conclude with a discussion exploring the potential of understanding OD as a fundamental human practice to reduce these power-related obstacles.

## Introduction

The implementation of Open Dialogue (OD) introduces changes on two different levels: First, a culture of dialogical communication between staff, users, and caregivers is supported, promoting open exchange, transparency in decision-making as well as favoring context-bound understandings over symptoms and clinical diagnostics. Second, community-based, multi-disciplinary teams are organized to offer primarily outpatient services: immediate help in crisis, continuity of support by the same team, a low and selective use of medication and a primarily psychotherapeutically oriented approach are key principles of OD, requiring major structural changes for their implementation in current mental health care systems (Olson et al., 2014).

Building on these therapeutic and organizational principles, adopting OD throws various “paradigmatic givens” of the mental health system into question, which may also lead to implementation obstacles. This tension has been dealt with in more depth in another essay (von Peter et al., 2021) and summarized in a more recent publication describing how the OD approach leads to “challenges between core clinical values, and conflicting expectations of professional practice and performance” (Lennon et al., 2022). As a result, the implementation of OD may “generate organizational, professional, and personal resistances” (Søndergaard, 2009), leading to major problems in its acceptance and adoption in practice. For this reason, it is important to examine what can modulate such resistances and how OD practitioners or trainers can actually contribute to enabling them or preventing them.

This perspective paper aims to reflect power relations as potential disruptive factors for the implementation of the OD approach in mental health care. It follows the main line of question as to which power emerges from us as OD practitioners and trainers, and how this power makes it more difficult to implement OD. For this purpose, we draw on data from a small implementation study carried out between 2017 and 2018, of which the main results are drawn upon elsewhere (Putman and Martindale, 2022). This study material is first briefly given as a background and then analyzed by the authors from different perspectives. Thereby, we first use rather open, associative reflections, as usually applied during the OD Reflecting Team OD practices (Olson et al., 2014), followed by a more structured discussion, exploring the potential of understanding OD as a fundamental human practice to reduce the power-related implementation obstacles described.

## Extracts of study material

The study mentioned was of an exploratory nature, aimed at understanding the doubts and in certain cases resistance of about 2/3 of the members of a training group to implement OD in their daily clinical practice after an extensive training of roughly one and a-half years. This training took place in a university hospital setting, involving trainees from diverse institutional backgrounds, including staff in day clinics and out-and inpatient facilities. Although the training program started with a significant group of approximately 25 persons attending, little by little, the attrition rate progressively climbed from session to session. Upon completion, only 5–8 persons continued to practice OD network meetings on a continuous basis, thus leading to the question of the need to understand the motivations behind the other participants’ decision not to engage in this approach.

12 problem-centered interviews were carried out with staff trainees from various occupational backgrounds, asking about the reasons for not further engaging in the OD approach. In most of the interviews, it quickly became clear, that power relations were at the center of what hinders the implementation process, a topic that therefore we have chosen to focus on here. The interview material was re-coded by one of the authors (SvP) to provide for an empirical basis for further reflections on this topic. Only a selection of the relevant passages could be represented in this manuscript due to reasons of space.

Faced with a multitude of definitions and varieties of conceptualization, power will be understood in the sense of Foucault’s notion of “capillary power,” conceptualizing it as a rather diffuse, generalized potency that plays out in every interaction and exchange and is spread throughout society, instead of using a more top-down definition that understands power as more direct force of domination or oppression (Foucault, 1975). To facilitate understanding, selected quotes from the interviews are given under sub-headings below and integrated within explanatory texts that are of a more interpretative nature. These verbatims are followed by further reflections by each author based on their experiences with OD prior to discussion.

### Power games

The interviewees made clear that the OD training polarized teams that had previously been working well together:

*“Unfortunately, our teams have been divided since the OD training. People first felt energized by the training -[…] but that only went so far, as power games came into play.”* (Interviewee (=I)2)

These power games were described as having emerged quite early on over a conflict of competence:

*“… the wrangling over therapeutic competence between the staff in training and the staff not being trained came into play right from the second training session onwards.”* (I3)

### Threat to power relations

This dynamic was perceived to result first of all from the very nature of the OD approach, described as threatening to traditional power relations:

“*The call to make yourself present, transparent, and authentic isn’t everybody’s cup of tea […] there is always the possibility of being confronted with your own mistakes and the shortcomings of the system. And there is the danger […} that your own expertise is no longer the most important.”* (I7)

And:

“*OD is incompatible with the current system that does not cherish controversy at all. Traditional structures demand clear definitions and orders… OD is much less hierarchical, more horizontal. […] Letting go of that power can be quite liberating, but also intimidating.”* (I8)

### Know it all

Second, these power dynamics were thought to derive from the particular behavior of the OD practicing staff, in particular from its perceived attitude to know everything better:

*“I think that there is a danger that people who practice OD see themselves as superior and this can be experienced as a provocation… as if in their infinite wisdom they know it all or they are better than other staff.”* (I9)

And:

*“The people who practice OD convey the idea that everything else aside OD is or has been wrong or worth less. They are on a mission to convince all the other staff to practice only OD.” (I6)*

### What are their motivations?

This behavior led to mistrust and accusations that the OD staff lacked transparency in terms of their motivation:

*“Sometimes, it is not clear to me, what do these OD people want? What are they up to? What is their goal?”* (I2)

And:

*“…they claim that everything is possible – for instance using the notion of polyphony or rejecting hierarchies – but actually there is a hard and fast line of what is allowed and what is not.”* (I6)

## Reflections of the authors

To increase the polyphony of interpretation, in the following, the empirical material is reflected upon by the three authors of this manuscript, providing for various ideas, images, feelings, and associations that have arisen, when reading and discussing the empirical material. This form of “inner dialogues” or “reflective talk” is frequently used during practicing OD (Olson et al., 2014), meant to use various styles and rhetoric with the goal to elicit multiple viewpoints and to escape the risk of too monolithic interpretations *via* an assemblage of emergent thoughts and divergent associations. In the discussion part, these reflections will be integrated into a more coherent narrative that reflexively deals with possible solutions to reducing the power-related implementation obstacles described.

### Sebastian

I want to begin my reflection by describing a recent experience: I went to an OD community center to check its suitability as a research center. Normally I am very critical when I visit clinical facilities. Here the opposite was the case: I was open, felt at home, was in direct contact with the staff. After this visit I felt bad, and I did not know why. Only gradually, I realized that during this visit, I had functioned as part of an idealized community.

While I usually keep a critical distance from psychiatric services of any kind, I felt fully immersed in this situation without any “ifs and buts”: I rather freely related to my OD colleagues, checked their attitude much less critically than in other situations and used fewer precautions to protect myself. Coming from a heavy Nazi background on both sides of my family, such an immersion had quite a personal impact on me, inciting warnings not to engage too much in any form of ideological circles, which only gradually became comprehensible to me.

Certainly, this story is heavily related to my own history and my resulting perspective on this world. Yet at the same time, the above quotes make clear that the implementation of OD is linked to a powerful demarcation of an OD social identity (apparently also perceptible from the “outside” as well), leading to rigid outside-inside boundaries: following the described implementation process, the teams *“have been divided,”* whereas those who practice OD saw *“themselves as superior”* and “*everything else aside OD”* was described by them as being “*wrong or worth less,”* drawing a rather “*hard and fast line of what is allowed and what is not.”*

Thus, apparently a rather rigid social identity has been developed among those that had been trained in OD, for which I will use, for didactic reasons – with the intention to elicit strong reactions that often help to clarify arguments – the strong metaphor of a “sect.” This social identity has led to the perception of an in-and outside of this group of trained professionals, leading to various questions such as: is the OD community a rather rigid community, binding us together in the form of an ideologically charged grouping, perhaps with strengthening our feelings of connectedness and solidarity, but certainly at a cost? Is such an inflexible grouping useful, given the difficulties that usually occur when a new intervention is implemented in the field of mental health care, or does it not rather make implementation processes more strenuous, thus being certainly of no interest to those that want to practice OD?

With this in mind, a huge number of further questions arise – some of which have also been discussed during a conference, at which the provocative question of “in how far does the OD community resemble a sect?” had been discussed vividly: is there one single OD community, and if so, who are “we”? Do we share certain intentions and what are our motivations? Even more, are we following a “mission,” for instance to combat the medical system or to reform our society, and is there only one mission, or inversely, do we really allow for polyphony both within our community and in relation to the outside? Which (implicit) moral or ethical messages are we acting out when practicing or providing training in OD? And finally: is the OD approach, and are we, who practice or train people in it, as power reflexive as it/we claim(s) to be?

And further: given its principles, should the primary task of the OD approach not be to allow for a plurality and diversity of voices? Do not we make ourselves untrustworthy if we openly or implicitly devalue other mental health care practices or ways of thinking? What about the principle of multi-vocality in this case? How can we allow for difference and open exchange between different approaches in the field of mental health care, without giving-up or watering down our own principles or achievements? Even more so as it increasingly seems to be difficult in this world to exchange views across different positions to create an understanding for each other. Self-contained perspectives or communities are not helpful in this context but may rather reinforce harmful identity politics.

At the same time, during this conference, it became clear that the image of a sect is a powerful one, thus also raising various concerns among the discussants: does this image lead to the OD approach being perceived to be less scientific? Does it foster a stereotype of the OD community as an entangled coterie, or does it lead to constructive discussions about its implicit or explicit exclusion mechanisms or power relationships? In short, does it do more harm in relation to OD training, implementation, or advocacy or does it rather lead to more transparency and a greater acceptance of this approach?

### Katrin

I do not think there’s anything wrong with having an ethic. And we are creating spaces together with others. These others are our fellow human beings and colleagues. So, we are a group, and this inevitably involves a social identity. Yet, right from the start, in trainings, we can engage in open dialogue, allowing for more polyphony. I think that is possible. We all have different experiences. That is a gift.

“Teaching means learning,” a simple sentence. I think this means to respect and give space for everyone during the learning and teaching process, to enable multiple viewpoints as an antidote to power. Further, may learning the practice through the practice itself help to diminish power differentials? Almost the whole conference in Tornio took place in a dialogical way. In Finland, they have an outstandingly good school system also, in which dialogue is practiced and taught. I wonder if such a “flow-in-action” may be related to the traditions of the Finnish Sami people? As we all know, there are techniques, even for witchcraft. And what we call “witchcraft” – or a “sect” – might be a common, old practice between us humans: the urge to gather, share feelings, ideas, stories.

The past is the present: in each society, there seems to be an urge to normalize misdeeds, traumata, and violence. Collective memories, experiences that crisscross families, such as wars, institutionalized violence, child abuse, etc. (Psychiatric) institutions were part of these horrors. It feels to me that in German psychiatry, there is an unbroken tradition since the Nazi times. Not directly, but the much earlier death of the “mentally ill” (also due to treatment (Wunderink et al., 2013; Begemann et al., 2020)) seems to be widely accepted. Given this context, how can we implement OD without passing on or acting out power?

Maybe, these contextual understandings should be more reflected: should we open-up more spaces for these stories in the OD trainings and network meetings and also in society? A lot of people working in psychiatry have a lot to share. And if we teach and moderate and try to build up precious spaces (pedagogical flow? witchcraft?), we must ask ourselves whether we can bear these horrors done to our people, fellow travelers, or maybe to us. And we also must ask ourselves whether we project our understandable fears or feelings of these horrors onto others. To not feel the pain ourselves while working. Remaining simple and compassionate is a big thing. And small at the same time. All we can do. A small thing…

### Katharina

The interview data makes one clear: listening to people who do not choose to use OD after being trained is vital to be able to learn how to pass it on successfully. One goal of OD is to empower people regardless of their position in a network, so the question of how power is perceived and dealt with during processes of OD implementation is a central one too.

I was surprised that OD trainers were perceived as “Knowing-it-all,” because one of the OD principles is “tolerating uncertainty,” which to me seems to be the opposite of being in a knowing position. In my understanding, while developing OD in Tornio, they did not introduce a set of principles to change a running service or to form a new one. Instead, a continuous self-reflective research approach was started. Thus, the methods of practice were continuously improved and powerful “us versus them” distinctions between practitioners were avoided. Further, I heard from Finnish teachers, that OD cannot be taught, but can only be learned. It is not about doing something to someone, but creating opportunities for curiosity, dialogue, learning, which the so called “student” can freely choose to make use of or not.

The part about threat to power relations particularly resonated with me. One reason why I value Open Dialogue is because of its different perception of who is in charge, seeing each network member as a living being who is responsible for their own process.

If we feel safe, we are more eager to try out new things and more able to access our prefrontal cortex (Porges, 2011). Notions of “power games” or “non-transparent motivation” may point to a lack of safe space during trainings or in work situations. Maybe certain preconditions that allow participants to show vulnerability or curiosity have not been fulfilled, which to me seems to be quite often the case in a medical and hierarchical environment where “the doctor is always right.”

This hierarchical organization in hospitals may have detrimental consequences: If you have not been listened to or been devalued several times, you become careful and will no longer answer questions openly or expose your critical positions. For instance, I am thinking of nurses, psychologists, medical interns, or patients who traditionally were not supposed to question a doctor’s decision. Being part of these powerful hierarchical relationships has even influenced me, a doctor that *via* her role usually is perceived as being on the rather sunny side of the system’s hierarchy, while I often did not feel powerful at all: from some colleagues’ derogative comments about others, I feared that if I showed insecurity or doubt about my work, I would be talked about in a similar way. Even when I felt overwhelmed, I tried not to show it.

It took me years to allow myself to feel and express my pain and insecurity in certain situations – for example, about coercive measures I prescribed from lack of better alternatives in my context -and I am not yet finished with that issue either. Several group settings with colleagues both in OD and other contexts helped me to find my voice and agency. Only through this can I now learn to improve how I treat patients in these situations. Without that support, I would not have had the strength to further pursue my career or OD.

## Discussion

From the above reflections, the question arises as to what can be done to reduce the power-related implementation obstacles described? One of the most frequent questions arising in discussion about the OD approach has to do with whether this approach is an intervention/method/technique, or rather – and thus seeing the issue as a simple, raw dichotomy – an attitude toward life/position/culture? What are the power-related consequences of each of these positions, when practicing, speaking about, or disseminating the OD approach? Are there any dangers or pitfalls if a practitioner decides in favor of one of them? To reflexively deal with power differentials, we advise the second option: thus, it may be good to remember that the OD approach makes use of a human being’s abilities and need to think and act dialogically. OD is making use of this basic cultural practice, it makes attempts to create a space for it. Such a perception of OD may provide a more modest, less powerful, and simpler view of this approach: OD is nothing special but has reached out to enact a fundamental human practice that we all share if we (dare to) practice it.

At the same time, perceiving OD as a cultural practice could call into question the very notion of implementation: is it truly possible to “implement” Open Dialogue? Is “implementation” the right word when it comes to (re-?)learning or further developing the basic human ability of dialogically relating to each other (see also 10)? We all have experiences with (non-)dialogical interactions and conversations, within both our private and professional lives. Certainly, the mental health systems we work in usually do not provide for sufficient possibilities to practice dialogical forms of care. Even worse, current systems may require the opposite: to speak and act monologically. Is it the actual dialogue that needs to be “implemented” or should we instead create a favorable context so that dialogical work becomes possible – in the sense of enabling it – an environment that is certainly worthy of insisting we make happen? Focusing on the context rather than on the nature of a social group or belonging, the latter view may balance or reduce powerful processes of identity politics and, thus, contribute to a solution in dealing with them.

At the same time, insisting on creating such an enabling environment may have the potential too to result in power struggles, leading to the next possibility to constructively deal with power differentials, when implementing the OD approach: Even if psychiatry may have its own dogma, this does not entitle us to (ab)use OD to create a counter-ideology. Thus, we should be careful with disseminating unifying or unified messages. Instead, we should allow for the dialogue between different “versions” of OD, accept contradictions and ambivalences, as well deal openly with the risk of monological preaching or dissemination of this approach: critique of OD ideology could be included in trainings and enabling practices as an integral part that is always present. This is even more important in the case of “top-down” implementation (the boss wants OD, and the employees must implement it), thus opening-up the opposite question of how we can find better means to enable OD bottom-up?

Seen this way – and this may be a further way out of the described power struggles –, any forms of dialogical teaching, communicating, and disseminating the OD approach are helpful to prevent dogmatism in relation to the implementation of the OD approach. The perception of OD as a basic cultural practice provides us with a guiding image here, to be used in connection with related processes of training and dissemination. This is even more important as this approach raises fundamental questions in relation to psychiatry, entailing the danger of too certain, all-embracing, monological answers to human suffering and existence. Instead, how can we create dialogical, meaningful, open, and safe spaces for doubt and skepticism that at the same time make positive experiences with the OD approach accessible and understandable to others? How can we transfer and debate knowledge without becoming (overly) monological, or closing-down variance and difference?

While discussing our perspectives, a critical study on feminist women’s groups of the 1970s came to our minds (Freeman, 1971): in the beginning, many of these groups avoided any leadership or directives for political reasons, with a devastating consequence: implicit power relations could expand and stabilize, often being more difficult to identify than authoritarian control. Thus, claiming “openness,” “tolerance,” or “polyphony” will not suffice to make power visible in OD spaces. Quite the contrary, these affirmations can be abused as effective weapons or invitations to powerfully occupy them. Thus, a continuous reflexivity appears necessary to better understand what emanates from us when we practice or enable OD, how we position ourselves in relation to our community/ies and toward “the” outside(s).

When discussed at the conference, the image of a “sect” seemed to dominate huge parts of its closing session, making clear how powerful it is, thereby foreclosing or reducing possibilities of alternate interpretations and investigation. Likewise, during the writing process, we recurrently wiggled with power issues, such as falling short of sufficiently reflecting on questions, such as: who sets the topic, who invites whom for which reflection, who is in editorial power, and whose contributions are adapted to which scientific and academic contingencies? As a result, achieving a dialogue between each author through their contributions has not been easy. In this sense, even thinking and writing about power struggles may itself be fueled with power. But maybe, it is naïve to believe that human interaction would ever be free of interests and different ways of asserting interests. As if writing or speaking against power will make you run the risk of falling into its trap.

## Data availability statement

The original contributions presented in the study are included in the article/supplementary material, further inquiries can be directed to the corresponding author.

## Author contributions

SP has developed the first draft. KS and KE have added their reflexions and ideas to the discussion section as well as revised the manuscript many times. All three authors have contributed to the draft of the attached manuscript. All authors contributed to the article and approved the submitted version.

## Funding

Funded by the Brandenburg Medical School Theodor Fontane (Medizinische Hochschule Brandenburg Theodor Fontane, MHB) publication fund supported by the German Research Foundation (Deutsche Forschungsgemeinschaft, DFG).

## Conflict of interest

The authors declare that the research was conducted in the absence of any commercial or financial relationships that could be construed as a potential conflict of interest.

## Publisher’s note

All claims expressed in this article are solely those of the authors and do not necessarily represent those of their affiliated organizations, or those of the publisher, the editors and the reviewers. Any product that may be evaluated in this article, or claim that may be made by its manufacturer, is not guaranteed or endorsed by the publisher.
